# HIV continuum of care: expanding scope beyond a cross-sectional view to include time analysis: a systematic review

**DOI:** 10.1186/s12889-021-11747-z

**Published:** 2021-09-17

**Authors:** Georgia Vourli, Ioannis Katsarolis, Nikos Pantazis, Giota Touloumi

**Affiliations:** 1 Department of Hygiene, Epidemiology & Medical Statistics, Medical School, National and Kapodistrian University of Athens, M. Asias 75, 115 27 Athens, Greece; 2 Medical Affairs, Gilead Sciences Hellas and Cyprus, Elliniko, Greece

**Keywords:** HIV, Continuum of care, Cross-sectional, Time analysis, Review

## Abstract

**Introduction:**

The continuum of care (CoC) model has been used to describe the main pillars of HIV care. This study aims to systematically review methods and elucidate gaps in the CoC analyses, especially in terms of the timing of the progression through steps, recognized nowadays as a critical parameter for an effective response to the epidemic.

**Methods:**

A PubMed and EMBASE databases search up to December 2019 resulted in 1918 articles, of which 209 were included in this review; 84 studies presented in major HIV conferences were also included. Studies that did not provide explicit definitions, modelling studies and those reporting only on metrics for subpopulations or factors affecting a CoC stage were excluded. Included articles reported results on 1 to 6 CoC stages.

**Results:**

Percentage treated and virally suppressed was reported in 78%, percentage diagnosed and retained in care in 58%, percentage linked to care in 54% and PLHIV in 36% of the articles. Information for all stages was provided in 23 studies. Only 6 articles use novel CoC estimates:

One presents a dynamic CoC based on multistate analysis techniques, two base their time-to-next-stage estimates on a risk estimation method based on the cumulative incidence function, weighted for confounding and censoring and three studies estimated the HIV infection time based on mathematical modelling.

**Conclusion:**

A limited number of studies provide elaborated time analyses of the CoC. Although time analyses lack the straightforward interpretation of the cross-sectional CoC, they provide valuable insights for the timely response to the HIV epidemic. A future goal would be to develop a model that retains the simplicity of the cross-sectional CoC but also incorporates timing between stages.

**Supplementary Information:**

The online version contains supplementary material available at 10.1186/s12889-021-11747-z.

## Introduction

The primary goal of HIV care is durable viral suppression, as there is solid evidence that virally suppressed individuals have zero probability of transmitting HIV [1, 2] and reduced mortality and morbidity [3]. Despite the great achievements of modern antiretroviral therapy (ART) in terms of viral suppression, HIV infection remains a major public health threat worldwide [4]. The steps leading to viral suppression, such as early diagnosis and secure linkage to and sustained retention in care are of great importance [5–8], underlining the need of monitoring the whole spectrum of HIV care [5]. The cascade or continuum of care (CoC) model has been proposed as a tool to illustrate the sequential steps or stages of HIV care pathway that people living with HIV (PLHIV) go through, from infection to initial diagnosis, ART initiation and viral suppression, subsequently expanded to also include linkage to and retention in care. Typically, from its first description, CoC presents a snapshot of the proportion of PLHIV who are successfully managed at each stage of care [5].

Aiming to eliminate HIV globally and taking into account a holistic approach to HIV care, UNAIDS had set the 90–90-90 target by 2020: 90% of all PLHIV to be diagnosed; 90% of those diagnosed to be on antiretroviral treatment (ART); and 90% of those on ART to become virally suppressed [9]. The UNAIDS target was updated to 95–95-95 by 2030 [10]. Monitoring of the UNAIDS targets can be evaluated using a 4-stage, cross-sectional CoC, assessed at a particular time point and in a particular country and displayed in a simple and easily interpretable figure [5].

A cross-sectional CoC is useful for monitoring public health goals and evaluating the performance of HIV care programmes, mostly because of its simplicity, straightforward interpretation and easy construction. Nevertheless, comparisons between different settings or evaluation of progress through time require presentation of common stages, estimated using unified definitions [11]. Data sources used for the estimation of CoC can also affect the outcome and should also be considered when comparing results of different studies [12].

Apart from the variability among cross-sectional CoC in the number and definitions of stages, cross-sectional analyses lack the dimension of time, which in the case of HIV can greatly affect patients’ and programmes’ outcomes. It has been shown that the time from seroconversion to diagnosis is crucial, since individuals at an early stage of infection who are not yet on ART have a higher probability of transmitting HIV [13]. Moreover, late presentation to care (currently defined in terms of the CD4 count at diagnosis being below 350 cells/μl or of a clinical AIDS diagnosis within 3 months of HIV diagnosis) is prevalent in most European countries and is associated with adverse short and long-term clinical outcomes, and with protracted transmissibility [14]. Similarly, the time from diagnosis to ART initiation is crucial for both increasing the chance of retention in care and decreasing the risk of further transmission [3]. Other important measures to evaluate HIV care include the time PLHIV remain in care after their initial engagement and the time to AIDS onset or other important clinical events. A significant time-bound parameter is the time of sustained undetectability (also defined as durable suppression). Ignoring the time component, cross-sectional CoC metrics may overestimate health care achievements [15]; even in countries where the 90–90-90 target has been achieved, late presentation to care is still common [16], while modelling studies indicate that the target of zero new infections will not be achieved. Indeed, in the UK, It has been shown that for men who have sex with men (MSM), providing immediate ART at diagnosis and assuming constant rate of condomless sex, an increase of up to 90% in the proportion of virally suppressed PLHIV within 1 year of infection is needed for a reduction in HIV incidence from 6/1000 to 1/1000 person-years [17]. Similarly, in Australia, it has been estimated that achievement of the 90–90-90 target would only lead to a 10% reduction in incidence by 2020. Even with 95–95-95, intensified testing and condom use for all MSM, and 100% PrEP coverage for high-risk MSM, the maximum reduction in incidence by 2030 would be only 80% [18].

Considering the importance of monitoring HIV care and the potential challenges associated with the CoC design and data sources, we performed a systematical review of the literature on the HIV CoC and identified methods used so far. We aimed to provide a summary of the literature in terms of both the number of stages presented and their definitions, with a focus on whether and how often the timing of progression across stages is considered along with the corresponding metrics used. Ultimately, we aimed to provide a guide to researchers by describing methods used so far along with their strengths and limitations. We also provide guidance for future extensions to the CoC.

## Methods

We searched the PubMed and EMBASE databases up to the end of 2019 using the query ((((Cascade [Title/Abstract]) OR Continuum [Title/Abstract]) OR 90–90-90[Title/Abstract]) AND care [Title/Abstract]) AND (HIV [Title/Abstract] OR AIDS [Title/Abstract]). The term “90–90-90” was included to ensure that studies that specifically aimed on assessing the UNAIDS HIV targets would be selected. Studies presented in international HIV/AIDS conferences (EACS, CROI, HIV Drug Therapy Glasgow, IAS) during the last 3 years (2017–2019) were also reviewed. Since our aim was to describe methods used in original HIV CoC research, studies of other publication type (i.e. reviews, comments and editorials) were not included in the analysis.

**Fig. 1:**
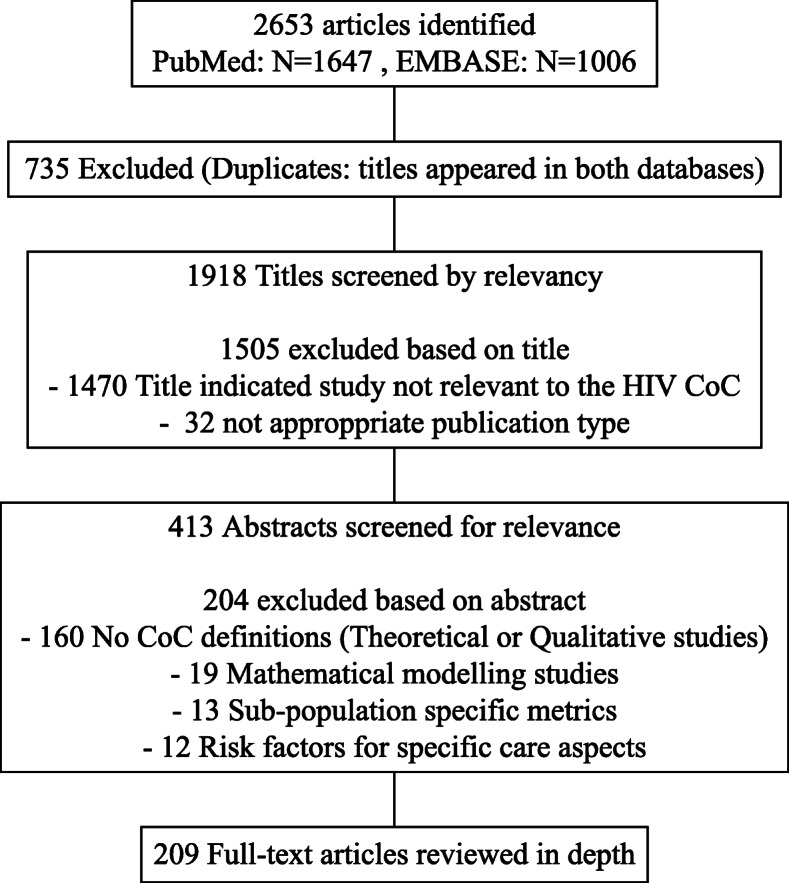
Full-text article selection process

Because of the nature of our search terms and the wide interest for the HIV CoC, especially during recent years, the search resulted in a large number of studies. The steps of the review process are presented in Fig. 1. In the first step, results from PubMed and EMBASE were merged and duplicate studies were excluded. Then, two authors (GV and IK) compared the list of titles published as full articles to those presented in a conference and in cases where studies were presented in a conference and subsequently published in a journal, the former were excluded. In the next step, full articles were separately analysed from conference presentations, following identical steps. The final list of unique titles was then reviewed. Studies in which the title clearly indicated that they did not report results on the HIV CoC were excluded from further analysis. The abstracts of the remained studies were then reviewed. Reasons for excluding a study at this point included a) being a qualitative study, b) not providing CoC definitions, c) being solely a mathematical modelling/projection study, d) focusing on indices of care specifically meant for subgroups and e) aiming to identify risk factors of dropping-out of one or more stages of the CoC rather than describing the CoC per se. The next step required reviewing the full text of the publications.

**Fig. 2:**
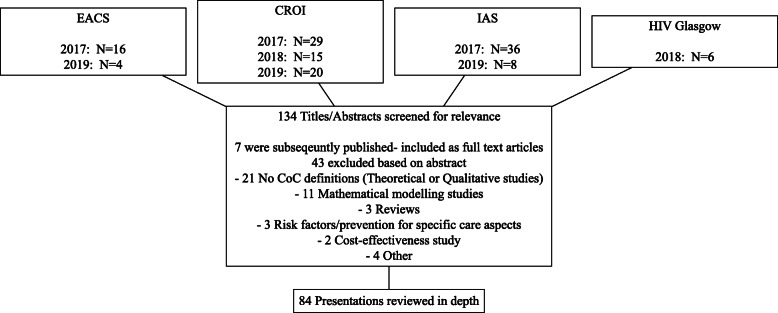
Conference presentations’ selection process

A similar process was followed for the conference presentations. Study titles were screened for relevance through the conference search engine. In cases where a search engine was not available, the abstract book (both title and abstract) was screened manually. At the final step, the poster or the presentation’s slides were reviewed. Selection criteria for further review were the same as those used for the full publication articles (Fig. 2). To assess the accuracy of this process, two reviewers (GV and IK) independently reviewed a random list of 100 articles and reached a very good agreement between them (Cohen’s k = 0.93). Articles beyond the random list that were of similar nature to the ones on which the two reviewers originally disagreed were then identified. Inclusion or exclusion of these articles was decided based on a consensus reached after further discussions between them.

## Results

In total 209 full text articles were included (Supplement, Table 1). They reported results on a spectrum of minimum 1 and maximum 6 CoC stages, i.e. total number of people living with HIV (PLHIV) including diagnosed and undiagnosed population, those diagnosed, linked to and/or retained in care, PLHIV treated with antiretrovirals and those virally suppressed (VS). Table 1 provides details on the data sources and the definitions of the CoC stages included in the reviewed articles.

**Table 1:** Reported Continuum of Care stages and corresponding definitions in the 209 full-text reviewed studies

Stage	Data source	Definition
**PLHIV** (*N* = 76, 1st stage in 76 of them)		
29	Surveillance	Estimated by mathematical modeling (ECDC modeling tool^a^, Spectrum^b^ or other)
45	Survey	Individuals testing positive, previously diagnosed or undiagnosed
2	Cohort	Estimated by mathematical modeling (ECDC modeling tool, Spectrum or other)
**Diagnosed** (*N* = 120, 1st stage in 67 of them)		
51	Surveillance	
67	Survey	Self reported (66), confirmed by reviewing medical records (1)
2	Estimated	
**Linked** (*N* = 170, 1st stage in 66 of them)		
34	Surveillance	Examined at least once (31) or in specific time interval after diagnosis (2), Other (1)
76	Cohort	Considered by definition linked as cohort participant (58), Examined at least once (17), Other (1)
53	Survey	Self reported (31), Examined at least once (19) or in specific time interval (1), Other (2)
7	Other	As Reported by participating countries (1), Health/social insurance records (2), cohort and survey data (1), comparison of multiple definitions-sources (1), Laboratory database (1), medical records (2)
**Retained** (*N* = 120, 1st stage in 1 of them)		
28	Surveillance	Examined at least once (4) or in specific time interval (23) after linkage, Other (1)
57	Cohort	Examined at least once (22) or at specific interval(s) (31) after linkage, Considered retained as cohort participant (3), Other (1)
32	Survey	Self reported (8), Examined at least once (6) or in specific time interval (18) after linkage
3	Other	Examined at least once (1) or in specific time interval (2) after linkage
**Treatment** (*N* = 163, 1st stage in 6 of them)		
16	Surveillance	Medical records: Ever on ART (12), Currently on ART (4)
82	Cohort	Medical records: Ever (60), Currently (13), Initiated in specific interval from diagnosis (6), Time spent on ART (1), Non-specified (2)
53	Survey	Self reported: Ever (11), Current (37), Adherence (5)
12	Other (multiple: 5, countries estimates: 4, Lab database: 2, RCT: 1)	Ever (7), Current (5)
**Viral Suppression** (*N* = 162)		
47	Surveillance	Medical records (47)
78	Cohort	Medical records (78)
35	Survey	Self reported (16), Laboratory test result in the context of the survey (18), Combination of information from medical records and exams during the study (1)
2	Clinical Trials	Examination meant for the study

^a^ECDC HIV modelling tool can be downloaded from https://www.ecdc.europa.eu/en/publications-data/hiv-modelling-tool

^b^Spectrum can be downloaded from www.avenirhealth.org

The included articles provide CoC estimates for the period 2007–2018. Most of them (134, 64.1%) concern on subpopulations of individuals. Specifically, 53 articles report estimates for one or more key populations (MSM, people who inject drugs (PWID), transgender women, ethnic groups, marginalized individuals, migrants, refugees, sex workers (female or male), young/adolescents/children and women). In the remaining 81 studies, samples included individuals diagnosed either in a specific time interval or area. Details can be found in the Supplementary Table 1.

In 26 articles (12.4%) national (Australia, Brazil, China, France, Estonia, Georgia, Greece, Haiti, Italy, Japan, Oman, Singapore, South Africa, Sweden, Switzerland, and USA) or multi-national (Europe and Central Asia, European Union, South America, Sweden and Denmark) CoC estimates are provided while in 21 of these the total number of PLHIV in the country/region are reported, based on mathematical modelling (details can be found in the Supplementary Table 2). In 23 out of these 26 articles, the main data source was national surveillance systems, supplemented by other data sources, mainly cohorts in 12 articles; Estimates were based on nationwide surveys or cohorts only in the remaining 3 articles.

**Fig. 3:**
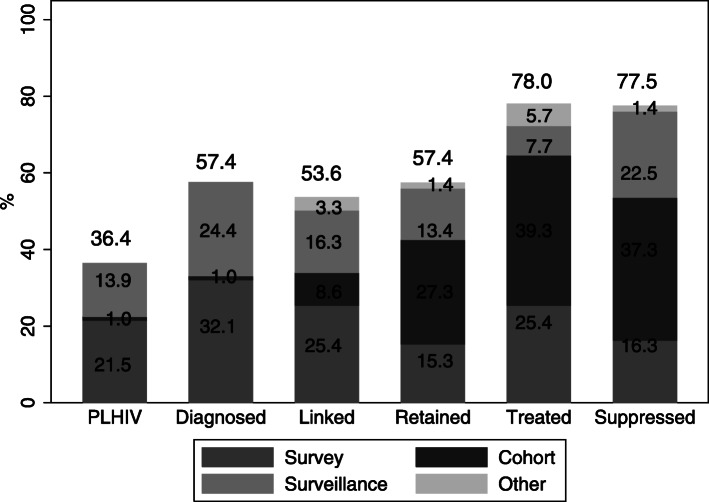
The percentage of articles providing estimates for each CoC stage is shown above each bar. Inside the bars, the percentages of data sources used to produce estimates of each stage is presented

In total, 76 (36.4%) articles estimated the undiagnosed fraction of PLHIV. Thirty-one of these studies reported estimated number of PLHIV, applying mathematical models on surveillance data (29 studies) or other estimation methods on key population or non-nationwide cohort data (2 studies). In 45 studies the number of PLHIV was not reported, but the fraction of undiagnosed PLHIV was estimated as the number of those non-previously diagnosed over the total number of individuals found to be HIV positive when tested in the context of the study (Table 1, Fig. 3).

One hundred and twenty articles (57.5%) reported either the number of diagnosed individuals or the percentage diagnosed among HIV positive individuals. Specifically, in 67 of these articles, the percentage of diagnosed individuals was estimated as the number of individuals who reported they already were aware of their status over the total number of HIV positive survey participants. In one of them, HIV status was confirmed reviewing medical records. Fifty-one articles provided the number of diagnosed individuals as the one reported by national surveillance systems, and 2 more studies provided nation- or region-wide estimates of the number of diagnosed individuals (Table 1, Fig. 3).

Fifty-eight articles reporting data from cohorts were considered as starting from the step of HIV linkage to care onwards, although most of them did not explicitly identify this important step as such. One-hundred and twelve articles explored linkage to care: linkage was self-reported in 53 articles; in 34, linkage was determined based on medical record review, while in 20 articles cohort participants were considered linked only if further criteria were fulfilled (Fig. 3). In 5 articles linkage was determined based on other data sources (health insurance records 2 articles, multiple data sources and laboratory results 2 studies). Regardless of the data source, in 67 articles all individuals with one physical contact with HIV health services (i.e. one visit or examination) were considered as linked to care. In only 3 articles this contact was required to be in a specific interval after diagnosis for an individual to be considered as linked (Table 1). One hundred and twenty articles reported on retention to care based on data from surveys (32), cohorts (57), surveillance (28) or other sources (3) (Table 1, Fig. 3). Retention was defined as one contact with health services after linkage in 33 articles, while in 74 studies, this contact was required to be in a specific interval after linkage (Table 1). In addition to the standard CoC measures, 21 articles reported on the timing of the engagement to care, i.e. linkage and/or retention, such as the time to engagement, percentage of person-time retained in care, or factors affecting time to linkage and loss to follow up.

Overall, 163 studies reported on ART as a stage of the CoC, most of them exploiting cohort data (Table 1, Fig. 3). Most of the studies defined treatment as having ever initiated ART, while 59 articles defined it as current use of treatment. Furthermore, 6 studies examined prompt treatment initiation, i.e. within a specific time interval from diagnosis. Only adherence to treatment (rather than initiation) was considered in 5 of the included studies, while in 2 studies the definition of treatment was unclear (Table 1). Twelve of these studies provided extra information on the time to ART initiation and 4 of them explored factors associated with ART initiation, eligibility or prescription (Supplement, Table 2).

Viral suppression rates were reported in 162 studies. Medical records were reviewed in 124 articles, based on either cohort or surveillance data. In 16 articles viral suppression was self-reported, whereas viral load was measured in the context of the CoC study per se in 18 cases. In 2 cases, viral suppression was evaluated via laboratory testing in the context of clinical trials, while in one study information from medical records and laboratory test results were combined to evaluate VS (Table 1, Fig. 3). Four of the 162 studies reported time to VS, 2 of them reporting on factors affecting the time to VS and one also reporting risk factors for the time to virologic rebound. The threshold to determine VS ranged from 20 copies/ml to 1000 copies/ml. However, in most articles (77 out of 162) viral suppression was defined as less than 200 copies/ml.

Regarding the 84 conference presentations, 5 reported only on retention and 1 only on the frequency of VS evaluation. Thirty studies presented a 4-point CoC, with 21 studies reporting on PLHIV, diagnosed fraction, use of ART, and VS in compliance with the 90–90-90 UNAIDS target. Two studies following the 4-point CoC reported on linkage, retention or both. Only 4 studies presented results on time components of the CoC (comparison of cross-sectional CoC in different calendar years (2), comparison of the proportions of individuals at different times from diagnosis to ART initiation and at different CD4 classes at diagnosis cross-sectionally, estimation of the times from diagnosis to care enrolment, to ART initiation, and to virologic suppression).

Overall, 53 articles acknowledged the dimension of time. In 6 articles Cox regression was applied to estimate the effect of risk factors on the time to death (4), loss-to-follow-up i.e. duration of retention (3), loss of viral suppression (3) and ART initiation (2). Most of these articles evaluated more than one outcome having as time origin the date of diagnosis or the date of ART initiation. Eight studies applied Poisson regression to evaluate either temporal changes or differences on specific CoC stages’ rates among groups of individuals. The outcome was the ART uptake rate in 5, viral suppression rate in 5 and retention in care rate in 4 of these studies. These studies, although acknowledged the dimension of time, they did not provide any estimate of time-related measures (i.e. time intervals or incidence), but rather they focused on comparisons.

On the other hand, 7 articles that explored factors affecting rates or times (2 using Poisson and 5 Cox or Fine and Gray regression models) also provided summaries of the observed time between stages. Further, 8 studies provided summaries of observed time-to, rates of and time spent in a CoC stage, but ignoring censoring, i.e. not estimating them with time-to-event methods.

The Kaplan-Meier method was the most commonly applied method, used to estimate time-to one or more CoC stages in 17 articles, along with Cox models in 13 of them. The most common outcomes evaluated were time to linkage, ART initiation, loss-to-follow-up or viral suppression, measured either from diagnosis or from linkage to care.

Apart from the well-established time-to-event methods, six studies used novel methods to estimate the timing of the continuum of care. One of these studies described the dynamic patterns of care and retention using multistate methodology. In 3 articles the date of infection and time intervals from this time point to subsequent HIV care stages were estimated. Finally, two articles applied a recently developed method to estimate both time spent between stages and within each stage based on the estimation of the risk as derived from the cumulative incidence function, weighted for confounding and censoring (Table 2). A detailed presentation of all studies that included time related results is included in the Supplement (Supplementary Table 3).

**Table 2:** Summary of the 6 studies which present novel methods to estimate the CoC timing

Article	Time-related Method
Lee H et al. AIDS, 2018 [19]	The authors presented the dynamic patterns of care and retention using multistate methodology (Method described in: Lee H, et al., A state transition framework for patient-level modeling of engagement and retention in HIV care using longitudinal cohort data. Statistics in Medicine, 2017). After defining the CoC states, the transition probabilities between them were estimated using multinomial regression. Then, state membership (i.e. the probability of being in a state) was estimated as a function of the transition probabilities up to this state. In cases where endogeneous covariates had to be taken into account, e.g.disease markers, a joint model of the distribution of the covariate and of the state transition processes was applied to predict state membership probabilities.
Lesko CR et al. AIDS, 2016 [20] and Desir FA et al. Clin Infect Dis, 2019 [21]	In this article, the non-parametric estimates of the cumulative incidence were used. The investigators follow a method (described in: Cole SR, Lau B, Eron JJ, et al. Estimation of the standardized risk difference and ratio in a competing risks framework: application to injection drug use and progression to AIDS after initiation of antiretroviral therapy. Am J Epidemiol. 2015; 181 (4):238–45) for the estimation of risk based directly on the survival function. Rather than estimating hazards and hazard ratios, the method proposed the application of censoring and exposure weights to the Nelson-Aalen estimate of the cumulative incidence function.
Reyes-Uruena JM et al. BMJ Open, 2018 [22]	To estimate the annual incidence and the time of infection, the authors used the ECDC Modelling tool. The method is based on a multistate back-calculation model that estimates HIV annual incidence along the size of the undiagnosed population, using data on reported HIV and AIDS cases, and information on CD4 count at the time of diagnosis
Robertson MM et al. Clin Infect Dis, 2019 [23]	In this article the estimation of the time of infection was based on the CD4 cell count at diagnosis and the CD4 decline rate estimated by previous studies on seroconverters.
Supervie V et al. J Acquir Immune Defic Syndr, 2016 [24]	To estimate both the HIV incidence and the distribution of times from infection to diagnosis, the authors fitted a back-calculation model to the annual number of new HIV diagnoses (Ndawinz JD, Costagliola D, Supervie V. New method for estimating HIV incidence and time from infection to diagnosis using HIV surveillance data: results for France. AIDS. 2011;25:1905–1913). Nonparametric estimates of the cumulative distribution function for each stage were produced, accounting for censoring. Using the inversion method (Nonparametric Estimates of Cumulative Distribution Functions and Their Inverses. Available at: http://fr.mathworks.com/help/stats/examples/nonparametric-estimates-of-cumulative-distribution-functionsand-their-inverses.html?refresh=true), 10,000 random values for each time interval were generated. The estimated time intervals were summarised as means, medians and interquartile ranges.

## Discussion

HIV continuum of care attracts great interest, with major institutions issuing guidelines for its optimal presentation that would allow the assessment of the pre-determined care goals [9, 25, 26]. In this systematic review, we aimed to summarize methods applied in HIV CoC studies worldwide and report on their variability. Moreover, we investigated whether time parameters are included in the construction of the cascade model either as time between different stages or within each stage. Our results indicate that although a large number of articles reported on CoC measures, there is great heterogeneity among reported stages, definitions and data sources. Furthermore, only a small number of articles provided additional information beyond the standard, cross-sectional CoC, especially in relation to the time dimension.

The 209 included articles reported on at least 1 CoC stage. Most articles focused on ART and viral suppression (78.0 and 77.5% of the studies, respectively), as these may hold particular interest as crucial milestones for both public and individual health [1, 3, 27]. Linkage to and retention in care were estimated in more than half of the reviewed articles (53.6 and 57.4%, respectively). Although these measures cannot be used to evaluate the 90–90-90 target directly, they are prerequisites for both administration and adherence to ART and thus remain very relevant, even in the “test and treat” era [28]. On the other hand, only 36.4% of the articles provided estimation of either the number (14.8%) or the fraction (21.5%) of undiagnosed HIV population. According to previous studies, the undiagnosed fraction is the greatest weakness of the CoC [29]; a large undiagnosed fraction can lead to misleading conclusions regarding the success and public health impact of ART.

In more than half of the articles that provided estimates of linkage, it was defined as one contact (visit/examination) with health services, and few studies required this contact to be within a specific interval after diagnosis. On the other hand, retention was defined as a contact with health services in specific time intervals after linkage in most studies, but a considerable number of studies defined it as a contact with health services after linkage without further requirements. There are many articles, however, where both linkage and retention were self-reported without further clarifications. Differences in definitions between articles make comparison of results on the same stage among different studies infeasible. Similar issues existed in the definition of ART, as well. The majority of articles reported the proportion of individuals ever on ART, while several articles reported the proportion of individuals currently on ART, and a few studies reported only on adherence to ART. Having ever been on ART is considered a reasonable measure to capture the percentage of treated individuals, since ART is life-long treatment. Nevertheless, a non-negligible proportion of individuals are lost to follow-up after having initiated ART [30], thus failing to capture the actual follow-up status can lead to overestimating the second 90. Viral suppression is documented by medical records in most of the articles; however, a wide range of viral load thresholds (< 20 to < 1000 copies/ml) was used, complicating comparisons for this CoC stage, too.

Even in cases where the CoC stages are estimated using common definitions, differences in data sources hamper safe comparisons. Surveys, where outcomes are self-reported and cannot be verified by medical record reviews in most cases, potentially suffer from self-reporting bias. Ideally, the HIV CoC should be informed uniformly by monitoring national cohorts. However, national cohorts are lacking in most cases. Thus, data from cohorts which only include people linked to care are often used, which may result in biased estimates [12, 31]. A striking example is the estimation of the proportion treated among cohort participants, who are a selected sample of individuals who are diagnosed and linked to care. In these cases, the percentage treated may be on overestimate. This discrepancy may be even more pronounced for the key populations estimates, since previous studies indicate disparities in access to care within the population of PLHIV and thus uneven representation in the HIV cohorts [32].

It must be pointed out that surveillance systems are ideal data sources when the focus is on estimating the CoC of the general population or of subgroups characterised by easy-to-collect information, such as gender, age or, at most, risk group. Nevertheless, surveillance data typically do not include occupational and/or behavioural information, (e.g. such involvement in sex work.). PWID may also be under-represented in surveillance data [32]. Given that HIV epidemic is concentrated in such key populations, estimation of CoC among them is of great importance but requires appropriate sampling techniques. Most of the reviewed studies that focused on vulnerable and hard to reach populations, recruited their participants using Respondent Driven Sampling [33]. This ensures inclusion of the greater part of the reference population and, given the analysis considers the study design, leads to generalizable estimates.

National CoC estimates were provided in 26 articles (12.4%), 23 of which used surveillance data. In cases where the surveillance registry includes data on all diagnoses from the start of the epidemic in the country, the number of diagnosed individuals is directly available. However, even in cases where diagnoses were not systematically reported until recently, estimation of the total number of diagnosed individuals in a country is feasible. In Italy the number of diagnosed and linked to care individuals was estimated by a national survey [24], while in France it was estimated as the sum of estimates of the number of individuals in care (based on the number registered in the general social insurance scheme and its coverage) and not in care (based on the percentage of newly diagnosed not engaged to care) [34].

Cross-sectional CoC are easy to construct and useful when aiming to assess public health targets, such as the UNAIDS 90–90-90. However, cross-sectional measures have been shown to have a delayed response to changes and to produce biased, overoptimistic results for the performance of health systems under specific conditions [15]. Moreover, given ART effectiveness, ongoing HIV transmission is associated with delays in HIV diagnosis and linkage to care, mainly attributed to late diagnosis [7], delayed ART initiation [33] and HIV care interruptions [6]. Ideal HIV care would ensure that the percentage of individuals engaged in each stage would be as close as possible to 100%, and the time between infection and diagnosis, diagnosis and ART initiation, and ART initiation to viral suppression would be as short as possible. As a result, this would maximize the duration of viral suppression and minimize the risk of transmission. A graph illustrating this is presented in the Supplement. Recent studies have highlighted the importance of investigating, in addition to the standard cross-sectional CoC, time spend in each of the CoC stages [19, 24, 34]. Among the reviewed articles, a relatively small number have recently taken efforts to construct longitudinal CoC, most of them focusing on the time to reaching the next CoC stages and risk factors for delays between stages (Supplementary Table). The most common time-to-event estimation method was the Kaplan-Meier method, used in 17 of the reviewed studies to provide estimates of time intervals and/or cumulative incidence for one or more CoC stages. Despite the well-established benefits of early entry to care, only a few articles provided estimates of the time of infection and thus of the time between infection and diagnosis and/or ART initiation [22–24]. Apart from studies that considered the transition probabilities as the percentage of individuals that pass to the next CoC stage among those participating to the previous one stage [21, 35], one study analysed the CoC as a dynamic process with distinct states that individuals could enter and leave more than once during their ﻿lifetime﻿ [22]. These considerations can improve HIV health care programmes by identifying the CoC stages at which individuals are most likely to leave care. Another time-related aspect examined in three of the reviewed studies that could be of value, especially from a public health point of view, was the cumulative time spent in each CoC stage [21, 34, 35].

Although these elaborated analyses of the CoC are very important, they usually miss the simplicity and straightforward interpretation of the cross-sectional CoC. Thus, a future goal would be to accurately evaluate progress towards HIV elimination using an updated CoC model that retains the simplicity of the standard cross-sectional CoC but also incorporates time between stages. This will help highlight gaps in the time-response to the HIV epidemic, an important step toward the ultimate goal of zero new HIV infections.

## Supplementary Information

**Additional file 1: Table S1.** Articles presenting Continuum of Care estimates for subgroups of individuals (*N* = 134). **Table S2.** Articles presenting national and international estimates of the conitnuum of care (*N* = 26). **Table S3.** Articles acknowledging the dimension of time (*N* = 53, 38 of them present estimates of cumulative time on a stage, incidence or time-to a CoC stage). Two of these are also included to the national CoC estimates table.

**Additional file 2.**

## Data Availability

All data generated or analysed during this study are included in this published article and its supplementary information files.
