# Supplementary material for: HIV continuum of care: expanding scope beyond a cross-sectional view to include time analysis: a systematic review
Source: BMC Public Health. 2021 Sep 17;21:1699. doi: 10.1186/s12889-021-11747-z (PMC8447660; doi:10.1186/s12889-021-11747-z)
Supplement: Supplementary file 2 — Additional file 2. [file 12889_2021_11747_MOESM2_ESM.pptx]

## Slide 1
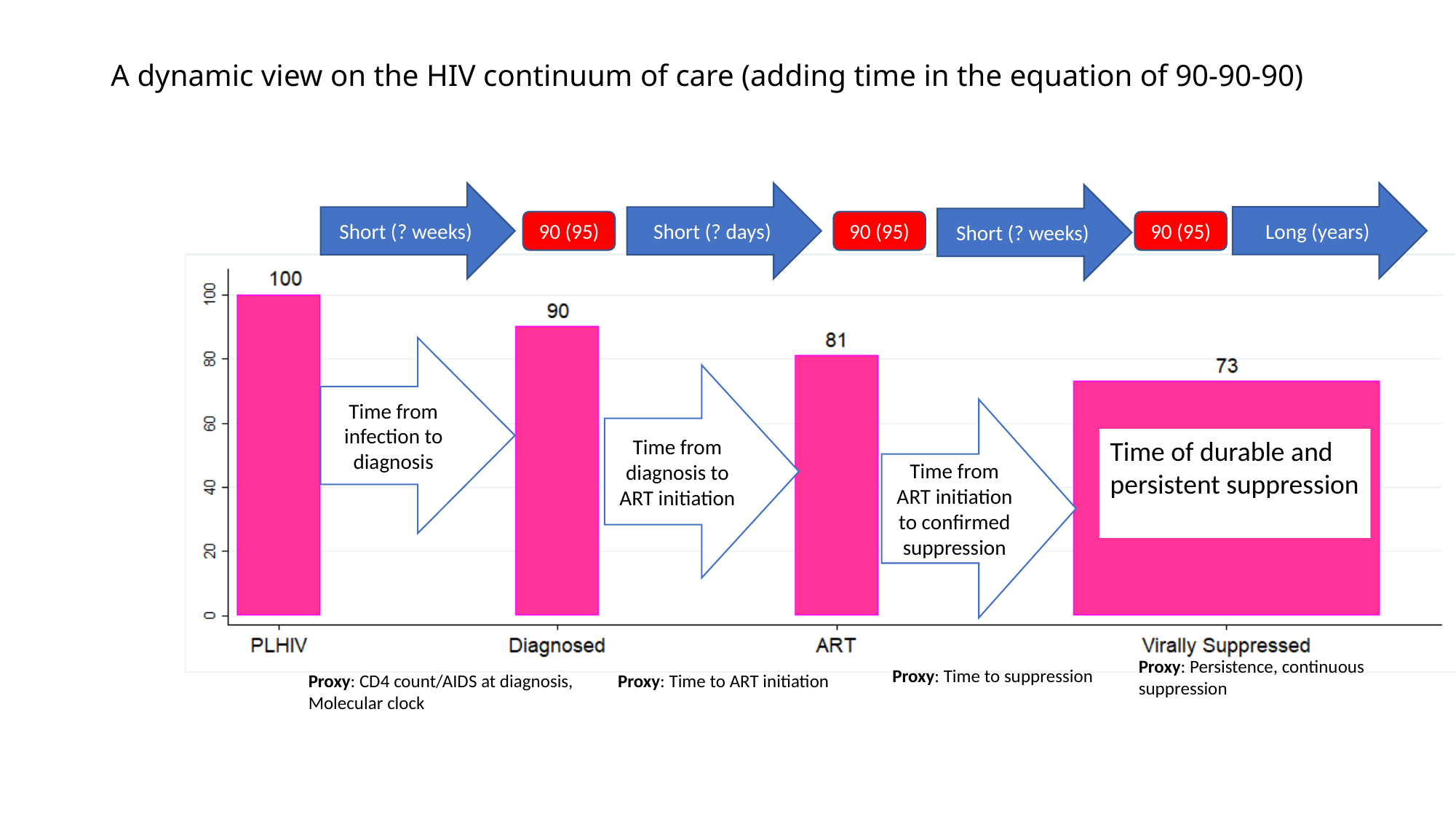

# A dynamic view on the HIV continuum of care (adding time in the equation of 90-90-90)
Long (years)
Short (? weeks)
Short (? days)
Short (? weeks)
90 (95)
90 (95)
90 (95)
Time from infection to diagnosis
Time from diagnosis to ART initiation
Time from ART initiation to confirmed suppression
Time of durable and persistent suppression
Proxy: Persistence, continuous suppression
Proxy: Time to suppression
Proxy: Time to ART initiation
Proxy: CD4 count/AIDS at diagnosis, Molecular clock
